# Assessing the impact of air pollutants on clinical visits for childhood allergic respiratory disease induced by house dust mite in Shanghai, China

**DOI:** 10.1186/s12931-022-01967-1

**Published:** 2022-03-05

**Authors:** Junyang Li, Yabin Hu, Huaiyuan Li, Yihang Lin, Shilu Tong, Youjin Li

**Affiliations:** 1 Department of Otolaryngology, Shanghai Children’s Medical Center, School of Medicine, Shanghai Jiao Tong University, 1678 Dongfang Rd, Pudong, Shanghai, 200127 China; 2 Department of Clinical Epidemiology and Biostatistics, Shanghai Children’s Medical Center, School of Medicine, Shanghai Jiao Tong University, 1678 Dongfang Rd, Pudong, Shanghai, 200127 China; 3 Department of Clinical Laboratory Medicine, Shanghai Children’s Medical Center, School of Medicine, Shanghai Jiao Tong University, Shanghai, China; 4 School of Public Health, Institute of Environment and Population Health, Anhui Medical University, Hefei, China; 5 Center for Global Health, School of Public Health, Nanjing Medical University, Nanjing, China; 6 School of Public Health and Social Work, Queensland University of Technology, Brisbane, Australia

**Keywords:** Air pollutants, Allergic respiratory diseases, Allergic respiratory diseases induced by house dust mite, Children, Clinical visits

## Abstract

**Background:**

The prevalence of allergic respiratory disease (ARD) is increasing worldwide during the last few decades, causing a great disease burden especially for children. Air pollution has been increasingly considered as a potential contributor to this trend, but its role in ARD induced by house dust mite (HDM-ARD) remains unclear, especially in time-series study.

**Methods:**

A positive reporting of respiratory allergy to named allergens was included by serum specific IgE testing. A time series Quasi-Poisson regression with distributed lag non-linear model, combined with generalized linear model was used to examine the effects of air pollutants on ARD, HDM-ARD and ARD induced by non-house dust mite (NHDM-ARD).

**Results:**

A total of 16,249 cases of ARD, including 8,719 HDM-ARD and 8,070 NHDM-ARD from 1 Jan 2013 to 31 Dec 2017 were involved in this study. Air pollutants were significantly associated with clinical visits for childhood ARD and HDM-ARD. Exposure to higher O_3_ and interquartile range (IQR) increment in O_3_ (40.6 µg/m^3^) increased the risks of clinical visits for childhood HDM-ARD (RR_lag0-5_ for the 95th percentile of O_3_: 1.26, 95% confidence interval (CI): 1.03, 1.55; RR_lag0-5_ for IQR increment (40.6 µg/m^3^): 1.09, 95% CI: 1.01, 1.17) and ARD (RR_lag0-5_ for the 95th percentile of O_3_: 1.19, 95% CI: 1.03, 1.38; RR_lag0-5_ for IQR increment (40.6 µg/m^3^): 1.06, 95% CI: 1.01, 1.12). In addition, higher O_3_ was associated with increased RR of boys with ARD (RR_lag0-5_ for the 95th percentile: 1.26, 95% CI: 1.05, 1.51; RR_lag0-5_ for IQR increment (40.6 µg/m^3^): 1.09, 95% CI: 1.02, 1.16) and HDM-ARD (RR_lag0-5_ for the 95th percentile: 1.36, 95% CI: 1.06, 1.75; RR_lag0-5_ for IQR increment (40.6 µg/m^3^): 1.11, 95% CI: 1.02, 1.22), but not in girls.

**Conclusions:**

Exposure to O_3_ appeared to be a trigger of clinical visits for childhood ARD, especially for HDM-ARD and boys. These findings provide novel evidence on the impact of air pollution on HDM-ARD, which may have significant implications for designing effective intervention programs to control and prevent childhood ARD, especially HDM-ARD, in China and other similar developing countries.

**Supplementary Information:**

The online version contains supplementary material available at 10.1186/s12931-022-01967-1.

## Background

Since the publication of the Allergic Rhinitis and its Impact on Asthma (ARIA) document in 2001 [1], the “one airway” concept has been accepted almost unanimously by the physicians to describe specific aspects of patients diagnosed with allergic rhinitis (AR) with or without allergic asthma (AA). The clinical phenotypes of AR and AA relevant to allergy are encompassed in the term “allergic respiratory disease” (ARD) and the concept of a united allergic airway reflects a shared underlying mechanism of pathogenesis. The increasing prevalence of ARD has been assessed by many epidemiological studies worldwide [2–4]. Importantly, environmental factors have been increasingly considered as potential major contributors to this trend [5, 6].

Although the exact pathogenesis of ARD remains unclear, the increased presence of outdoor air pollutants resulting from more intense energy consumption and exhaust emissions from cars and other vehicles, may play an important role in the development of ARD [7]. Air pollutants have been reported to be associated with worsening of ARD symptoms [8]. Nevertheless, allergens play a decisive role in the onset of symptoms and influence the clinical manifestations of ARD [9]. House dust mite (HDM) sensitization is a major causative factor in the development of ARD [10]. Furthermore, a study has revealed that HDM induced more severe late reactions than cat or pollens in asthmatic patients [11]. Components of the ultrafine fraction of particulate matter (PM) induce allergic pulmonary inflammation and act as adjuvant of the allergic response to HDM [12], which suggests that airway mucosal damage and impaired mucociliary clearance induced by air pollutants may facilitate the access of inhaled HDM to the cells of the immune system.

To the best of our knowledge, no study has addressed the effects of air pollution on ARD induced by HDM (HDM-ARD) and/or non-house dust mite (NHDM-ARD) to date. Therefore, in this study, we investigated the independent effects of air pollutants on ARD, HDM-ARD, and NHDM-ARD in Shanghai, China.

## Methods

### Study participants

Shanghai, located in the east of China (N30°40′-31°53′, E120°52′-122°12′), is the most populous city in China, has a subtropical monsoon climate with four distinct seasons. In this study, data on daily clinical visits (outpatient and inpatient visits) for childhood ARD, HDM-ARD and NHDM-ARD from 1 January 2013 to 31 December 2017 were collected from Shanghai Children’s Medical Center, the largest pediatric hospital affiliated to Shanghai Jiao Tong University School of Medicine. The outpatient visits include allergic respiratory diseases such as allergic rhinitis and asthma, etc. The inpatient visits include emergence department and general admissions for respiratory disease. The medical record included the age, gender, date of visit, total IgE level, all types of inhaled allergens (including HDM (Dermatophagoides pteronyssinus [Der p 1] and Dermatophagoides farinae [Der f 1]), cat/dog hair, molds, cockroaches, grass/tree and pollens) detection and identification. Principal diagnostic classification of childhood ARD was made according to the tenth version of the International Classification of Diseases for anaphylaxis due to asthma (ICD-10, J45-J46) and AR (ICD-10, J30). The participants should meet the following criteria: at least one inhaled allergen was positive and total IgE was above the normal range. The exclusion criteria were as follows: (1) children were negative for allergens detection; or (2) total IgE was normal; or (3) patients older than 18 years old.

### Assessment of allergens-specific IgE and total IgE in serum

Before testing, the performance qualification of total IgE had been done. The protocol had an inter-assay variance of 1.49% and an intra-assay variance of 1.63% when testing the low concentration (70 IU/mL) of quality control serum, and an inter-assay variance of 0.65% and an intra-assay variance of 1.65% when testing the high concentration (200 IU/mL) of quality control serum. Serum total IgE (TIgE) was determined quantitatively in all the patients using the BN II and BN ProSpec (N Latex IgE mono, Siemens, Germany), after calibration using commercial standard (also Siemens), and expressed in IU/mL. The TIgE reference range depends on the age of the individual [It ranges from 0 to 1.5 IU/mL in newborn infants, 0–15 IU/mL in infants during the first year of life, 0–60 IU/mL in children (1–5 years), 0–90 IU/mL in children (6–9 years), and 0–200 IU/mL in children (10–17 years)] [13]. HDM or HDM along with other more serum IgE species (sIgE) was found positive (≥ 0.35 IU/ml) by Western blotting using AllergyScreen™ human serum specific IgE allergen detection kit for specific inhalant allergens as described in previous publications [14, 15]. In this study, ARD induced by non-house dust mite such as cat/dog hair, molds, cockroaches, grass/tree and pollens was classified as NHDM-ARD.

### Air pollutants exposure assessment

**Fig. 1:**
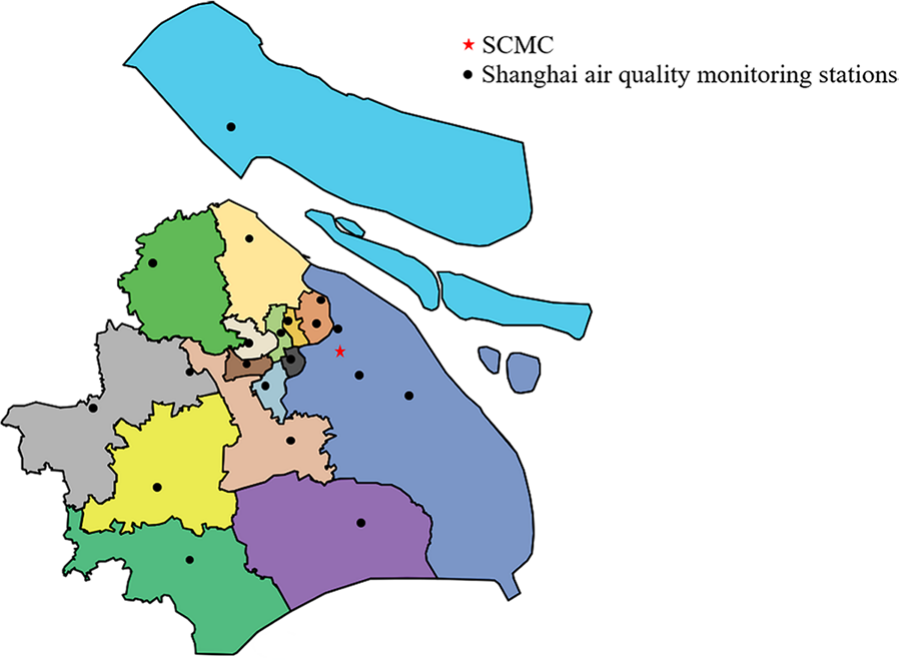
The locations of pediatric hospital and air quality monitoring stations in this study. SCMC: Shanghai Children’s Medical Center; Star represent the SCMC; Circles represent the monitoring stations

The data on air pollutants (μg/m^3^) were collected from the Shanghai Environmental Protection Agency, including nitrogen dioxide (NO_2_), sulfur dioxide (SO_2_), ozone (O_3_), and airborne particulate matter with an aerodynamic diameter less than 2.5 μm (PM_2.5_) or 10 μm (PM_10_). The meteorological data (daily mean temperature (Tmean, °C) was obtained from the Shanghai Meteorological Service. Daily mean values of air pollutants and meteorological factors were calculated using the 24-h monitoring records. Daily averages of air pollutants from many monitoring stations in various regions of Shanghai were used in this study (Fig. 1). Shanghai Meteorological Service is located at the center of the city, providing monitoring meteorological data with well calibrated and highly related to the records in other stations [16, 17]. In this study, we defined air pollutants as high level when the values were greater than the 95th percentile of each air pollutant.

### Statistical analysis

The analysis of the data was conducted in three phases. Firstly, we did descriptive analysis using mean, standard deviation (SD), interquartile range (IQR), minimum, maximum, 25th percentile (P_25_), 50th percentile (P_50_) and 75th percentile (P_75_) to describe daily clinical visits and environmental factors. Spearman’s correlation analysis was used to examine the correlations between environmental factors and clinical visits for childhood ARD, HDM-ARD and NHDM-ARD on the current day.

Secondly, a Quasi-Poisson generalized linear regression model combined with a distributed lag non-linear model (DLNM) [18] was used to determine the lagged and non-linear effects of air pollutants on childhood ARD, HDM-ARD and NHDM-ARD. Since Spearman correlation coefficients between NO_2_, SO_2_, PM_10_, and PM_2.5_ were high (r_s_ > 0.6), to avoid collinearity, only one of these variables was included in the final model, with the lowest Akaike information criterion (AIC) value. By comparing all multivariable models, we finally found that the model with Tmean and O_3_ performed the best, with the smallest AIC and residual deviance. The final multivariable model is as follows:

$$\mathrm{log }E(\mathrm{Y}t) =\mathrm{ \alpha }+\mathrm{ as}.\mathrm{factor}(\mathrm{dow}) +\mathrm{as}.\mathrm{factor}(\mathrm{holiday})+\mathrm{ ns}(\mathrm{time},\mathrm{df}/\mathrm{year}) +\mathrm{ cb}(\mathrm{Tmean},\mathrm{ maxlag}, 3\mathrm{df}) +\mathrm{ cb}(\mathrm{O}3,\mathrm{ maxlag}, 3\mathrm{df})$$

where *E* (Y_*t*_) is the number of daily clinical visits for childhood ARD, HDM-ARD or NHDM-ARD expected on day *t*; maxlag was set at 28 for temperature and 5 for O_3_ according to previous studies and the reference of AIC [19]; α is the intercept; day of the week (*dow*) and public holiday are controlled for as categorical variables; *ns*(*time, df/year*) is the natural cubic spline function (ns) for time (i.e., 1–1826 in total), with 9 *df/year* selected for the final model by calculating the minimum of the residuals using the partial autocorrelation function and based on the lowest AIC [20, 21]; *ns*(*time, df/year*) was used to control for seasonality and long-term trends in childhood ARD; *Tmean* indicates the mean temperature. *cb* represents the “cross-basis” function which defined the matrix about *temp, O*_*3*_ and lag using ns or linear function as appropriate.

Finally, sensitivity analyses were conducted to verify the robustness of the final results. We used 6–14 *df* per year for calendar time and 2–7 *df* for environmental factors in the model. The model included Tmean, PM_2.5_, and O_3_ were performed. We also set the maximum lag as 14 or 21 in the model to compare the results. We also did many subgroup analyses stratified by gender.

All analyses were conducted with R software 3.6.3. The statistical significance level was set at p-value < 0.05 (two-side).

### Ethics issue

The ethical approval of this project was granted by the Ethics Committee of Shanghai Children's Medical Center (approval number: SCMCIRB-Y2020100) prior to the data collection. Since the data were de-identified and aggregated, written consent was waived.

## Results

There were 16,249 ARD cases in total, including 8179 HDM-ARD and 8070 NHDM-ARD, of which there were 11,437 outpatients and 4812 inpatients from 1 Jan 2013 to 31 Dec 2017. HDM-ARD accounted for more than one half of ARD population (50.3%), and NHDM-ARD accounted for 49.7%. The number of outpatient visits was far greater than inpatient visits for either of childhood ARD, HDM-ARD and NHDM-ARD. The average age of daily clinical visits for childhood ARD, HDM-ARD and NHDM-ARD was 4.8, 5.6 and 3.9 years, respectively, in which boys made up the majority (62.7%, 64.0%, 61.4%, respectively). The median and IQR of TIgE value for childhood ARD, HDM-ARD and NHDM-ARD was 123.0 (48.4 ~ 278), 235.0 (118 ~ 574) and 77.1 (26.3 ~ 146) IU/ML, respectively (Table 1).

**Table1:** Characteristics of the patient episodes for ARD, HDM-ARD and NHDM-ARD

variables	ARD	HDM-ARD	NHDM-ARD	p-value
Total clinical visits	16,249	8179 (50.3%)	8070 (49.7%)	< 0.001
Outpatient visits	11,437 (70.4%)	6691(81.8%)	4746 (58.8%)	< 0.001
Inpatient visits	4812 (29.6%)	1488 (18.2%)	3324 (41.2%)	< 0.001
Age (years)				
Mean (SD)	4.8 (2.8)	5.6 (2.8)	3.9 (2.5)	< 0.001
≤ 2	2597 (16.0%)	436 (5.3%)	2161 (26.8%)	< 0.001
3–6	10,178 (62.6%)	5289 (64.7%)	4889 (60.6%)	
7–17	3474 (21.4%)	2454 (30.0%)	1020 (12.6%)	
Gender				
Male	10,193 (62.7%)	5238 (64.0%)	4955 (61.4%)	< 0.001
Female	6056 (37.3%)	2941 (36.0%)	3115 (38.6%)	
TIgE (IU/ML)				
Median, interquartile	123.0 (48.4 ~ 278)	235.0 (118 ~ 574)	77.1 (26.3 ~ 146)	< 0.001

*ARD* allergic respiratory disease, *HDM-ARD* allergic respiratory disease induced by house dust mite, *NHDM-ARD* allergic respiratory disease induced by non-house dust mite, *TIgE* serum total IgE. p-value was calculated by Pearson Chi-square test or Mann–Whitney U test

Additional file 1: Table S1 shows the summary statistics of air pollutants and daily clinical visits for childhood ARD, HDM-ARD and NHDM-ARD from 2013 to 2017. The median and IQR value of NO_2_, SO_2_, PM_10_, PM_2.5_, and O_3_ was 41 (30 ~ 56) μg/m^3^, 13 (10 ~ 19) μg/m^3^, 56 (40 ~ 82) μg/m^3^, 41 (26 ~ 63) μg/m^3^ and 72 (51 ~ 91.6) μg/m^3^, respectively. The daily median (IQR) number of clinical visits for childhood ARD, HDM-ARD and NHDM-ARD was 9 (5 ~ 13), 4 (2 ~ 7) and 4 (2 ~ 7), respectively. Additional file 1: Table S2 shows the associations of air pollutants and meteorological factor with childhood ARD, HDM-ARD and NHDM-ARD during 2013–2017. Spearman correlation coefficient between ARD and HDM-ARD was 0.88 (p < 0.05). There were positive associations of O_3_ with HDM-ARD (r_s_ = 0.08, p < 0.05), while no association was found for other air pollutants. Additional file 1: Figs. S1, S2 and S3 depict a time series plot of apparent long-term trends and seasonality of clinical visits for childhood ARD, HDM-ARD and NHDM-ARD, respectively. Additional file 1: Fig. S4 indicates the distribution of air pollutants during the period.

**Fig. 2:**
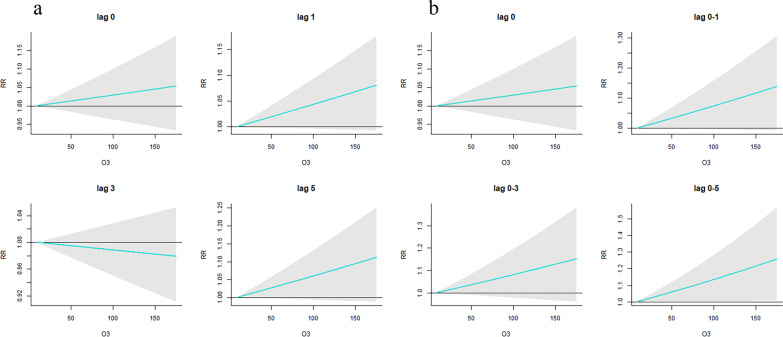
The single-day effects of O_3_ on the daily clinical visits for childhood ARD (**a**) and (**b**) the cumulative lagged effects of O_3_ on the daily clinical visits for childhood ARD. *RR* relative risk, *ARD* allergic respiratory disease; Green color indicates childhood ARD

Figure 2a shows that there was no statistically significant single-day effect of O_3_ on the daily clinical visits for childhood ARD. However, Fig. 2b reveals that higher O_3_ (relative risk (RR) _lag0-5_) was significantly associated with an increased risk of clinical visits for ARD in children.

**Fig. 3:**
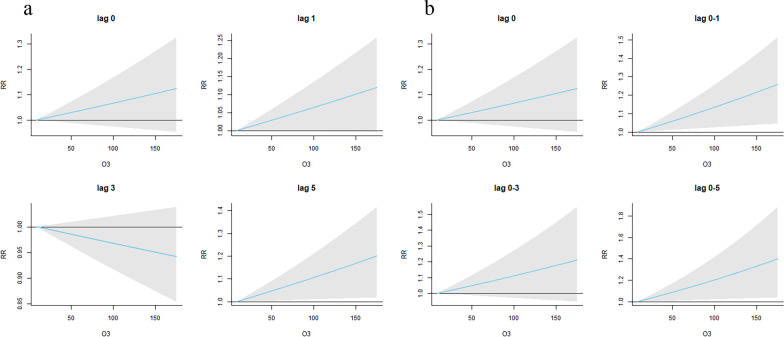
The single-day effects of O_3_ on the daily clinical visits for childhood HDM-ARD (**a**) and (**b**) the cumulative lagged effects of O_3_ on the daily clinical visits for childhood HDM-ARD. *RR* relative risk, *HDM-ARD* allergic respiratory disease induced by house dust mite; Blue color represents childhood HDM-ARD

Figure 3a indicates that the strongest relationship between O_3_ and childhood HDM-ARD was found at lag 5 days. Figure 3b depicts the cumulative lagged effects of O_3_ on the daily clinical visits for childhood HDM-ARD, suggesting that higher O_3_ (RR_lag0-1_ and RR_lag0-5_) was significantly associated with an increased risk of clinical visits for childhood HDM-ARD.

**Fig. 4:**
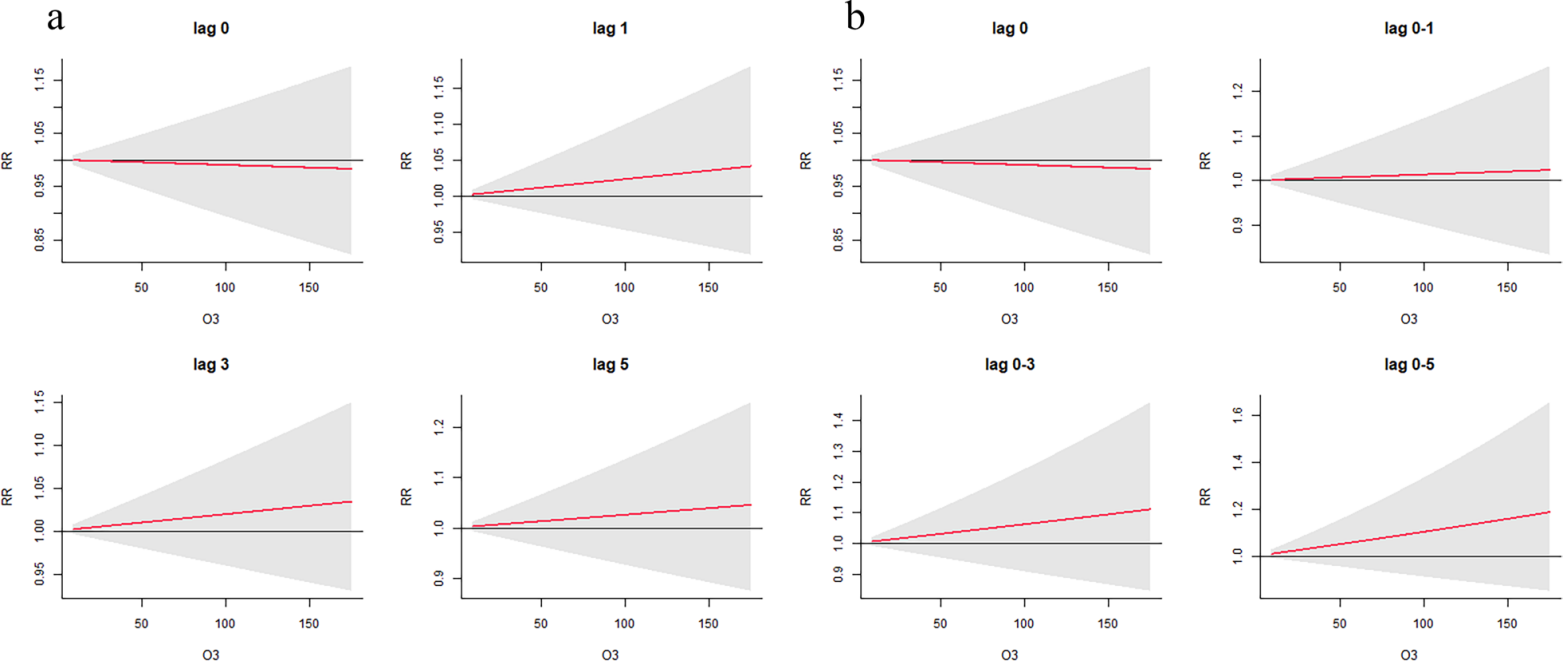
The single-day effects of O3 on the daily clinical visits for childhood NHDM-ARD (**a**) and the cumulative lagged effects of O_3_ on the daily clinical visits for childhood NHDM-ARD (**b**). *RR* relative risk, *NHDM-ARD* allergic respiratory disease induced by non-house dust mite; Red color means childhood NHDM-ARD

Figure 4a and b show the single-day effects and the cumulative lagged effects of O_3_ on the daily clinical visits for childhood NHDM-ARD, respectively. It indicates that O_3_ was not significantly associated with the risk of clinical visits for NHDM-ARD in children.

Additional file 1: Figs. S5 and S6 show the single-day effects and cumulative lagged effects of NO_2_, SO_2_, PM_10_ and PM_2.5_ on the daily clinical visits for ARD, HDM-ARD and NHDM-ARD in children, respectively. It suggested that NO_2_, SO_2_, PM_10_ and PM_2.5_ were not significantly associated with the risks of clinical visits for ARD, HDM-ARD and NHDM-ARD in children.

Table 2 shows the single-day effects and cumulative lagged effects of air pollutants on childhood ARD, HDM-ARD and NHDM-ARD after taking putative confounders into account. The cut points of 95th percentile of NO_2_, SO_2_, PM_10_, PM_2.5_ and O_3_ were 87.0 μg/m^3^, 39.0 μg/m^3^, 143.0 μg/m^3^, 118.0 μg/m^3^, and 123.0 μg/m^3^, respectively. Exposure to higher O_3_ (95th percentile, 123.0 μg/m^3^) or an IQR increment (40.6 μg/m^3^) elevated the RR of childhood ARD (RR_lag0-5_ = 1.19, 95% CI: 1.03, 1.38, and 1.06, 95% CI: 1.01, 1.12, respectively). However, the single-day effects of higher O_3_ and an IQR increment (40.6 μg/m^3^) were not significantly associated with the risk of clinical visits.

**Table 2:** The effects of air pollutants on daily clinical visits for childhood ARD, HDM-ARD and NHDM-ARD

Variables	ARD				HDM-ARD				NHDM-ARD			
	Lag (day)	Single-day effect	Lag (day)	Cumulative effect	Lag (day)	Single-day effect	Lag (day)	Cumulative effect	Lag (day)	Single-day effect	Lag (day)	Cumulative effect
High level												
NO_2_	2	1.02 (0.98, 1.07)	0–2	0.98 (0.88, 1.09)	2	1.04 (0.97, 1.11)	0–2	1.03 (0.88, 1.19)	5	1.07 (0.96, 1.20)	0–5	0.96 (0.78, 1.19)
SO_2_	5	1.02 (0.95, 1.11)	0–2	0.98 (0.89, 1.08)	5	1.04 (0.93, 1.17)	0–2	1.00(0.87, 1.15)	5	1.01 (0.91, 1.13)	0–5	0.99 (0.82, 1.20)
PM_10_	5	1.06 (0.99, 1.13)	0–5	1.04 (0.91, 1.18)	5	1.04 (0.94, 1.14)	0–2	1.02 (0.90, 1.16)	5	1.09 (0.99, 1.19)	0–5	1.05 (0.88, 1.26)
PM_2.5_	5	1.04 (0.98, 1.11)	0–5	1.06 (0.94,1.19)	5	1.04 (0.95, 1.14)	0–5	1.04 (0.87, 1.24)	5	1.04 (0.96, 1.14)	0–5	1.08 (0.91, 1.28)
O_3_	5	1.08 (1.00, 1.17)	**0–5**	**1.19 (1.03, 1.38)**	**5**	**1.13 (1.01, 1.27)**	**0–5**	**1.26(1.03, 1.55)**	2	1.03 (0.96, 1.12)	0–5	1.13 (0.89, 1.43)
IQR increase												
NO_2_	2	1.01 (0.99, 1.02)	0–2	0.99 (0.96, 1.03)	2	1.01 (0.99, 1.03)	0–2	1.01 (0.96, 1.06)	5	1.02 (0.99, 1.06)	0–5	0.99 (0.92, 1.06)
SO_2_	5	1.01 (0.99, 1.03)	0–2	1.00 (0.97, 1.03)	5	1.01 (0.98, 1.05)	0–2	1.00(0.96,1.04)	5	1.01(0.98,1.04)	0–5	1.00 (0.95, 1.06)
PM_10_	5	1.02 (1.00, 1.04)	0–5	1.01 (0.97, 1.05)	5	1.01 (0.98, 1.04)	0–2	1.01 (0.97, 1.05)	5	1.03 (1.00, 1.05)	0–5	1.02 (0.96, 1.07)
PM_2.5_	5	1.02 (0.99, 1.04)	0–5	1.02 (0.97, 1.06)	5	1.02 (0.99, 1.05)	0–5	1.01 (0.95, .07)	5	1.01 (0.98, 1.04)	0–5	1.02 (0.96, 1.08)
O_3_	5	1.03 (1.00, 1.06)	**0–5**	**1.06 (1.01, 1.12)**	**5**	**1.05 (1.00, 1.09)**	**0–5**	**1.09 (1.01, 1.17)**	2	1.01 (0.98, 1.05)	0–5	1.05 (0.96, 1.15)

Data were represented with relative risk (RR) and 95% confidence interval; bold value means statistically significant (p < 0.05);

The results were calculated with the 95th percentile of air pollutants (high level) or with IQR increase compared to the minimum value;

Single-day and cumulative effect reported the highest RR at a certain lag;

The final model, included the temperature, public holidays, day of the week, ns (time, df/year), and one air pollutant

For HDM-ARD, there were stronger cumulative lagged effects of O_3_ exposure (RR_lag0-5_ for the 95th percentile: 1.26, 95% CI: 1.03, 1.55; RR_lag0-5_ for IQR increment (40.6 μg/m^3^): 1.09, 95% CI: 1.01, 1.17) and the single-day effects (RR_lag5_ for the 95th percentile: 1.13, 95% CI: 1.01, 1.27; RR_lag5_ for IQR increment (40.6 μg/m^3^): 1.05, 95% CI: 1.00, 1.09, respectively).

For NHDM-ARD, neither the single-day effects nor cumulative lagged effects of O_3_ exposure were significantly associated with the risk of clinical visits.

**Fig. 5:**
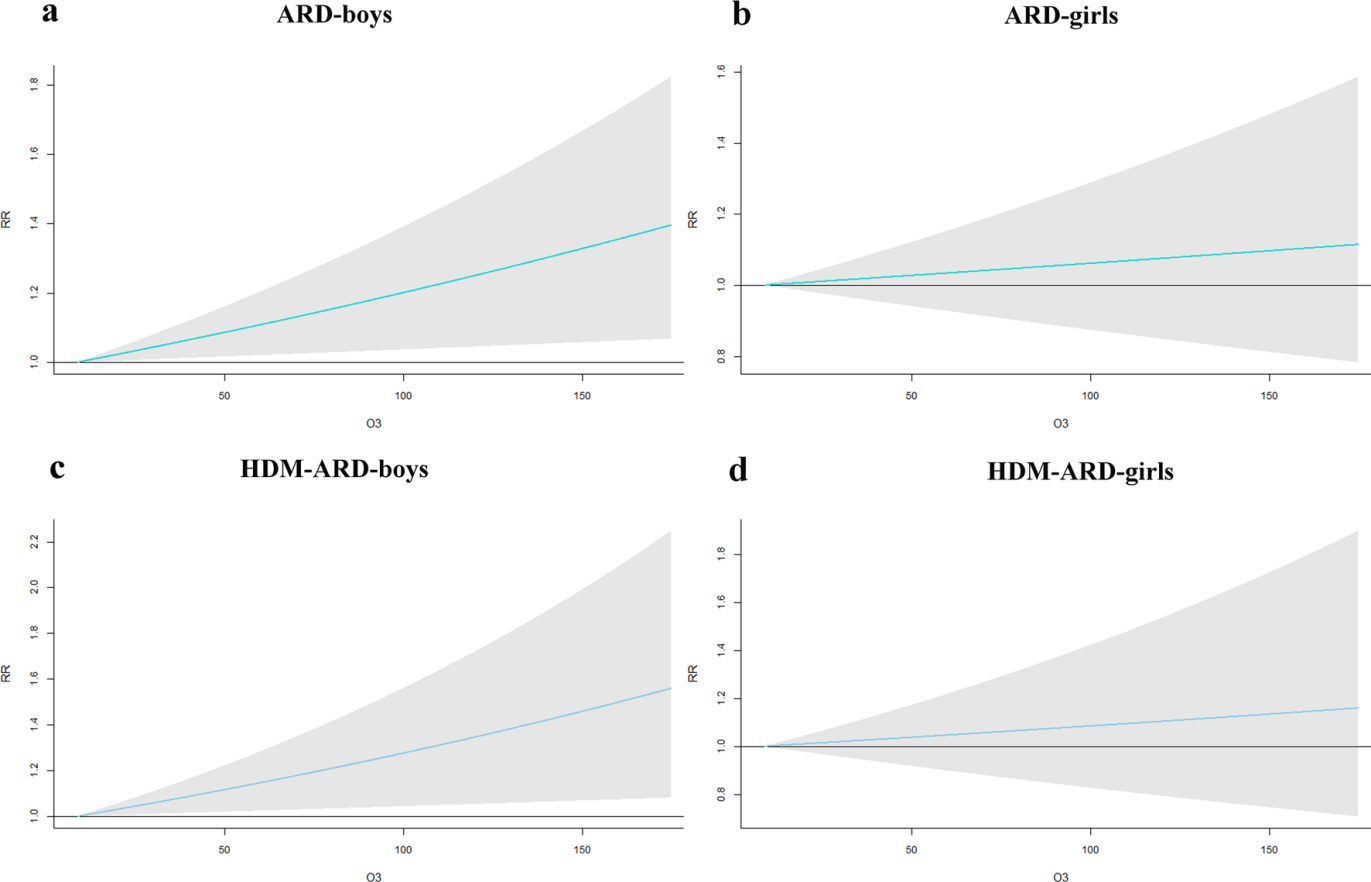
The overall effects of O_3_ on clinical visits of childhood ARD based on different genders (**a**, **b**). The overall effects of O_3_ on clinical visits of childhood HDM-ARD based on different genders (**c**, **d**). *RR* relative risk, *ARD* allergic respiratory disease, *HDM-ARD* allergic respiratory disease induced by house dust mite; Green color indicates childhood ARD; Blue color represents childhood HDM-ARD

Figure 5 shows the results of the stratification analysis based on different genders in O_3_. Higher O_3_ was associated with increased RR of boys with ARD (RR_lag0-5_ for the 95th percentile: 1.26, 95% CI: 1.05, 1.51; RR_lag0-5_ for IQR increment (40.6 μg/m^3^): 1.09, 95% CI: 1.02, 1.16) and HDM-ARD (RR_lag0-5_ for the 95th percentile: 1.36, 95% CI: 1.06, 1.75; RR_lag0-5_ for IQR increment (40.6 μg/m^3^): 1.11, 95% CI: 1.02, 1.22), but not in girls. Additional file 1: Fig. S7 shows the results of subgroup analysis by gender in other pollutants. But there was no significant association for other air pollutants.

Additional file 1: Fig. S8 shows the overall exposure–response relationships between exposure to O_3_ and clinical visits including outpatient and inpatient visits for ARD, HDM-ARD and NHDM-ARD. Additional file 1: Fig. S9 shows the overall exposure–response relationships between exposure to O_3_ and outpatient visits for ARD, HDM-ARD and NHDM-ARD. Additional file 1: Fig. S10 shows the overall exposure–response relationships between exposure to O_3_ and inpatient visits for ARD, HDM-ARD and NHDM-ARD. In the stratified analysis, for ARD and HDM-ARD, we found that O_3_ exposure was significantly associated with the increased risk of outpatient visits, while there was no such association for inpatient visits. There was no significant association between other air pollutants and inpatient or outpatient visits. In addition, the results show that both the single-day and cumulative exposures to O_3_ were significantly associated with the risk of clinical visits for the childhood HDM-ARD, whereas no such effect was found for childhood NHDM-ARD.

## Discussion

To the best of our knowledge, this is the first time-series study to examine the relationship between air pollution and childhood ARD induced by specific allergen such as HDM. The key findings of this study include: (a) O_3_ exposure was significantly associated with clinical visits for childhood ARD; (b) there was a stronger relationship between O_3_ exposure and clinical visits for childhood HDM-ARD; (c) the effects of O_3_ exposure on childhood ARD and HDM-ARD were markedly lagged; (d) in stratified analyses, a significant association was only found for outpatient visits but not for inpatient visits.

High level of O_3_ was associated with the risk of clinical visits for childhood ARD, particularly for HDM-ARD. Therefore, exposure to O_3_, might significantly increase the exacerbation of HDM-ARD in children and threaten their respiratory health. Air pollutants lead to increased mucosal permeability through airway inflammation in susceptible subjects, promoting inhaled allergen penetration and entry into immune system [22]. The role of pollutants in increasing ARD sensitization and symptoms has been reviewed elsewhere [23–27]. HDM—an important factor leading to ARD, is the main inhaled allergen in Shanghai [28]. Ye et al. found that haze facilitates sensitization to HDM in children [29]. A cohort study in Taiwan reported that children sensitized to HDM were most vulnerable to the adverse effects of air pollutants. In addition, HDM allergens may also alter the effects of air pollutants on ARD [9].

Although the mechanism underlying the relationship between air pollution and HDM-ARD was unclear, a review of mouse models and human studies suggests that the association might be mediated by an immune response [30]. Exposure high levels of air pollutants, particularly O_3_, PM, NO_2_ and diesel exhaust, could alter innate immunity. There is also evidence that components of air pollutants, particularly O_3_, diesel exhaust particles and total PM, interact with allergens in the air [31]. Due to this interaction, air pollutants can promote lung penetration of aeroallergens by increasing the release of allergenic proteins, leading to allergenic sensitization, and promoting Th2 inflammation and allergen-specific IgE response. Other studies have shown that HDM can directly or indirectly activate airway epithelial cells, leading to a variety of changes in allergic airway inflammation and the occurrence of HDM-ARD [32]. Epigenetic modifications induced by HDM reveal several changes in bronchial tissue that lead to inflammation and bronchial hyperresponsiveness. Furthermore, epigenome might influence susceptibility to mite sensitization by hypomethylation of the IL13 gene and DNA methylation in B-cell [33, 34].

In this study, we conducted stratified analyses to examine the effects of air pollutants on outpatient and inpatient visits for childhood ARD, HDM-ARD and NHDM-ARD. The results show that for ARD and HDM-ARD, we found that O_3_ exposure was significantly associated with the increased risk of outpatient visits, but no such association was observed for inpatient visits. No significant association was found for other air pollutants. The previous studies have not yielded consistent results on associations between O_3_ exposure and clinical visits or hospital admissions for ARD. For example, O_3_ exposure exacerbated asthma and increased the risk of asthma emergency department visits in the Seattle area [35]. However, a study in Taiwan found no association between O_3_ exposure and daily hospital admissions for respiratory conditions [9].

According to the stratification analysis of different gender, we found that gender was the factor influencing the correlation between air pollutants and ARD and HDM-ARD. Higher O_3_ was associated with increased RR of male children with ARD and HDM-ARD, but not in female children. Several studies have shown that the airways of male and female children respond differently to air pollutants [36, 37]. This is reasonable as there are differences in the airway between male and female children in the early and whole life stages of fetal lung development [38]. In childhood, the hyper-responsiveness of airway and ARD is more common among boys than girls [39]. As shown in this study, among the children, the stronger association between ambient O_3_ exposure and ARD and HDM-ARD was observed in males, which may be related to having less mature lungs and relatively narrower airways in boys than girls during childhood.

This study has four major strengths. First, this is the first time-series study to investigate the independent effects of air pollutants on childhood ARD, HDM-ARD and NHDM-ARD. Well-designed panel studies of time-series manner conducted in high risk (specific allergen sensitized) individuals could be sufficiently powered. Consistent with our findings, a cohort study on childhood environment and allergic diseases in Taiwan reported that children sensitized to HDM were most vulnerable to the adverse effects of air pollutants [9]. In addition, a large-scale cross-sectional study by Chen et al. found that O_3_ exposure may increase asthma exacerbation frequency [40]. Second, data from multi-sources including clinical records, air monitoring systems and meteorological services were integrated. Third, an advanced time series regression model (DLNM) was used in this study. The DLNM has increasingly been used in environmental health and epidemiological research. Finally, both single-day and cumulative effect estimates over 5 years were calculated to minimize short-term random variations.

Limitations of this study should also be acknowledged. Firstly, the cases in our study were selected from one hospital, and its generalizability may be limited. The findings of this study need to be interpreted with caution and multicenter studies are needed to validate these findings. Secondly, like other ecological time series studies, measurement bias is inevitable to some extent, since air pollution data were derived from monitoring stations, which could not be fully representative of individual exposures [41–43]. However, this type of measurement error may be non-differential, which may bias effect estimates towards the null [44]. Thirdly, some potential confounders that could affect the relationship between air pollution and ARD, such as influenza infections and life events [45], were not controlled for in this study because these data were unavailable.

To address the issues illustrated above, future research may focus on the following directions:

i. Prospective cohort studies are required to examine the causal/temporal relationship between environmental factors and childhood ARD, particularly HDM-ARD;
ii. Multi-center studies are needed to identify the influence of environmental factors on childhood ARD, HDM-ARD and NHDM-ARD;
iii. It is desirable to examine the interactive effects between air pollutants and meteorological factors on childhood ARD, HDM-ARD and NHDM-ARD;
iv. All potential confounding factors, including influenza infections and life events should be considered in further research.

## Conclusions

O_3_ exposure was significantly associated with the increase of clinical visits for childhood ARD, especially for HDM-ARD. These findings contribute to an in-depth understanding of the etiology of HDM-ARD, and suggest that it may be beneficial to adopt control measures (e.g., increased ventilation and mite removal) to avoid co-exposure to allergens and air pollutants. Moreover, these findings shed light on the impacts of air pollution on ARD, HDM-ARD and NHDM-ARD, which may have significant ramifications for designing effective intervention programs to control and prevent childhood ARD, especially HDM-ARD, in China and other developing countries around the world.

## Supplementary Information

**Additional file 1: Figure S1.** Time-series plots of clinical visits for childhood ARD for seasonality and trend from 2013 to 2017. **Figure S2.** Time-series plots of clinical visits for childhood HDM-ARD for seasonality and trend from 2013 to 2017. **Figure S3.** Time-series plots of clinical visits for childhood NHDM-ARD for seasonality and trend from 2013 to 2017. **Figure S4.** The distribution of air pollutants from 2013 to 2017. NO2: nitrogen dioxide; PM2.5: particulate matter less than 2.5 μm in aerodynamic diameter; O3: ozone; SO2: sulfur dioxide; PM10: particulate matter less than 10 μm in aerodynamic diameter. Blue smoothed lines were superimposed on each graph to present the long-term trends. **Figure S5.** The overall exposure–response association between NO2, SO2, PM10, PM2.5 and daily clinical visits for the single-day effects of childhood ARD, HDM-ARD and NHDM-ARD. RR: relative risk; ARD: allergic respiratory disease; HDM-ARD: allergic respiratory disease induced by house dust mite; NHDM-ARD: allergic respiratory disease induced by non-house dust mite; Green, blue and red color indicate childhood ARD, HDM-ARD, and NHDM-ARD, respectively. **Figure S6.** The overall exposure–response association between NO2, SO2, PM10, PM2.5 and daily clinical visits for the cumulative lagged effects of childhood ARD, HDM-ARD and NHDM-ARD. RR: relative risk; ARD: allergic respiratory disease; HDM-ARD: allergic respiratory disease induced by house dust mite; NHDM-ARD: allergic respiratory disease induced by non-house dust mite; Green, blue and red color indicate childhood ARD, HDM-ARD, and NHDM-ARD, respectively. **Figure S7.** The overall exposure–response association between NO2, SO2, PM10, PM2.5 and daily clinical visits for childhood ARD and HDM-ARD based on different genders. RR: relative risk; ARD: allergic respiratory disease; HDM-ARD: allergic respiratory disease induced by house dust mite; Green color indicates childhood ARD; Blue color represents childhood HDM-ARD. **Figure S8.** The overall exposure–response relationships between air pollutants and clinical visits for childhood ARD, HDM-ARD and NHDM-ARD. RR: relative risk; ARD: allergic respiratory disease; HDM-ARD: allergic respiratory disease induced by house dust mite; NHDM-ARD: allergic respiratory disease induced by non-house dust mite; Green, blue and red color indicate childhood ARD, HDM-ARD, and NHDM-ARD, respectively. **Figure S9.** The overall exposure–response relationships between air pollutants and outpatient visits for childhood ARD, HDM-ARD and NHDM-ARD. RR: relative risk; ARD: allergic respiratory disease; HDM-ARD: allergic respiratory disease induced by house dust mite; NHDM-ARD: allergic respiratory disease induced by non-house dust mite; Green, blue and red color indicate childhood ARD, HDM-ARD, and NHDM-ARD, respectively. **Figure S10.** The overall exposure–response relationships between air pollutants and inpatient visits for childhood ARD, HDM-ARD and NHDM-ARD. RR: relative risk; ARD: allergic respiratory disease; HDM-ARD: allergic respiratory disease induced by house dust mite; NHDM-ARD: allergic respiratory disease induced by non-house dust mite; Green, blue and red color indicate childhood ARD, HDM-ARD, and NHDM-ARD, respectively. **Table S1.** Distribution of daily clinical visits for childhood ARD, HDM-ARD, NHDM-ARD and air pollutants from 2013 to 2017. **Table S2.** Spearman correlation coefficients between environmental factors during 2013–2017.

## Data Availability

The data that support the findings in this study are available from the corresponding author upon reasonable request.

## Abbreviations

ARD: Allergic respiratory disease
HDM: House dust mite
HDM-ARD: Allergic respiratory disease induced by house dust mite
NHDM-ARD: Allergic respiratory disease induced by non-house dust mite
IQR: Interquartile range
ARIA: Allergic rhinitis and its impact on asthma
AR: Allergic rhinitis
AA: Allergic asthma
Der p 1: Dermatophagoides pteronyssinus
Der f 1: Dermatophagoides farinae
TIgE: Total IgE
SIgE: Serum IgE
NO_2_: Nitrogen dioxide
SO_2_: Sulfur dioxide
O_3_: Ozone
PM_2.5_: Airborne particulate matter with an aerodynamic diameter less than 2.5 μm
PM_10_: Airborne particulate matter with an aerodynamic diameter less than 10 μm
Tmean: Daily mean temperature
SD: Standard deviation
DLNM: Distributed lag non-linear model
AIC: Akaike information criterion
