# Supplementary material for: Assessing the impact of air pollutants on clinical visits for childhood allergic respiratory disease induced by house dust mite in Shanghai, China
Source: Respir Res. 2022 Mar 5;23:48. doi: 10.1186/s12931-022-01967-1 (PMC8897928; doi:10.1186/s12931-022-01967-1)
Supplement: Supplementary file 1 — Additional file 1: Figure S1. Time-series plots of clinical visits for childhood ARD for seasonality and trend from 2013 to 2017. Figure S2. Time-series plots of clinical visits for childhood HDM-ARD for seasonality and trend from 2013 to 2017. Figure S3. Time-series plots of clinical visits for childhood NHDM-ARD for seasonality and trend from 2013 to 2017. Figure S4. The distribution of air pollutants from 2013 to 2017. NO2: nitrogen dioxide; PM2.5: particulate matter less than 2.5 μm in aerodynamic diameter; O3: ozone; SO2: sulfur dioxide; PM10: particulate matter less than 10 μm in aerodynamic diameter. Blue smoothed lines were superimposed on each graph to present the long-term trends. Figure S5. The overall exposure–response association between NO2, SO2, PM10, PM2.5 and daily clinical visits for the single-day effects of childhood ARD, HDM-ARD and NHDM-ARD. RR: relative risk; ARD: allergic respiratory disease; HDM-ARD: allergic respiratory disease induced by house dust mite; NHDM-ARD: allergic respiratory disease induced by non-house dust mite; Green, blue and red color indicate childhood ARD, HDM-ARD, and NHDM-ARD, respectively. Figure S6. The overall exposure–response association between NO2, SO2, PM10, PM2.5 and daily clinical visits for the cumulative lagged effects of childhood ARD, HDM-ARD and NHDM-ARD. RR: relative risk; ARD: allergic respiratory disease; HDM-ARD: allergic respiratory disease induced by house dust mite; NHDM-ARD: allergic respiratory disease induced by non-house dust mite; Green, blue and red color indicate childhood ARD, HDM-ARD, and NHDM-ARD, respectively. Figure S7. The overall exposure–response association between NO2, SO2, PM10, PM2.5 and daily clinical visits for childhood ARD and HDM-ARD based on different genders. RR: relative risk; ARD: allergic respiratory disease; HDM-ARD: allergic respiratory disease induced by house dust mite; Green color indicates childhood ARD; Blue color represents childhood HDM-ARD. Figure S8. The overa [file 12931_2022_1967_MOESM1_ESM.docx]

**Supplementary Information for**: Assessing the impact of air pollutants on clinical visits for childhood allergic respiratory disease induced by house dust mite in Shanghai, China


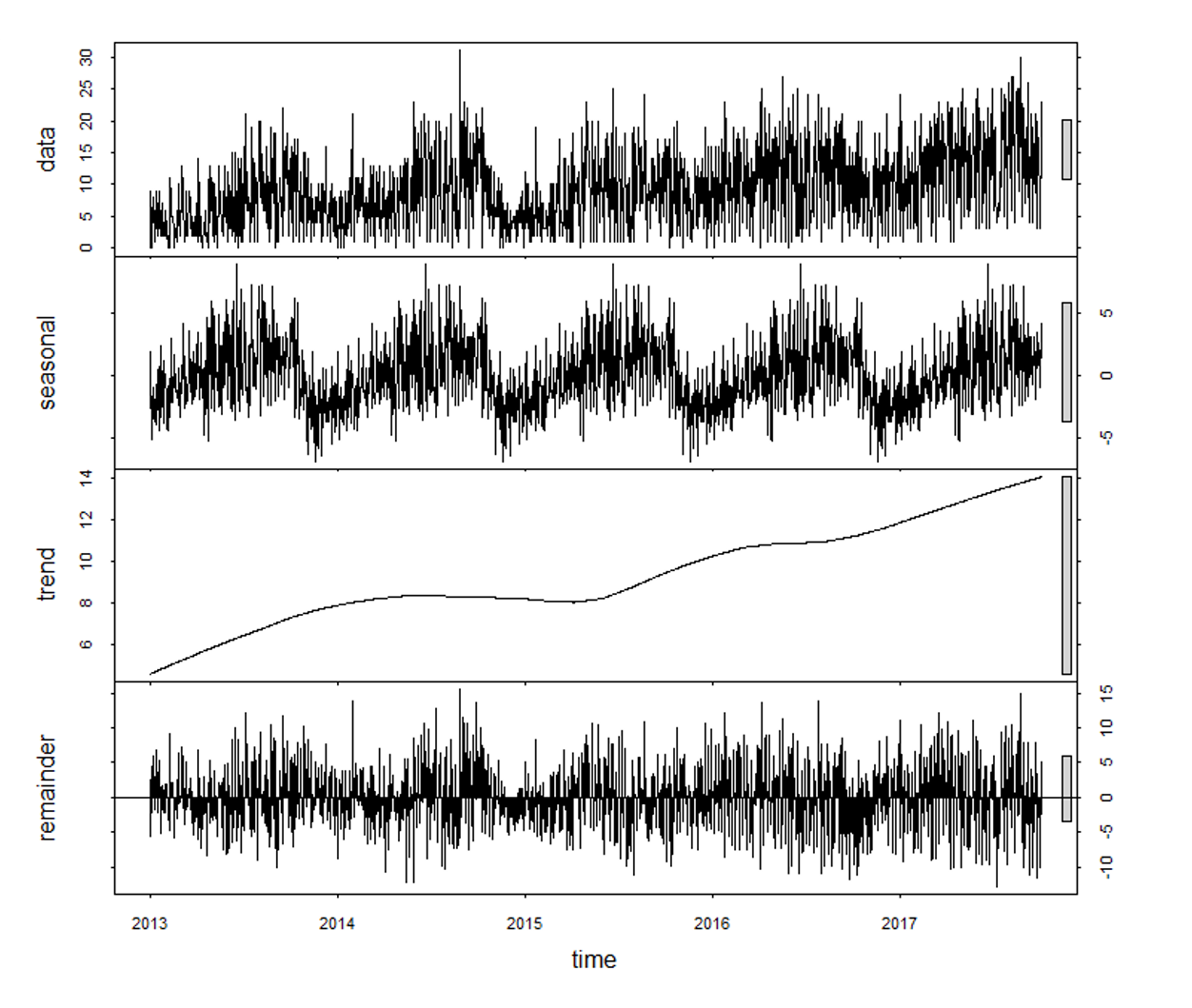


**Figure S1:** Time-series plots of clinical visits for childhood ARD for seasonality and trend from 2013 to 2017.


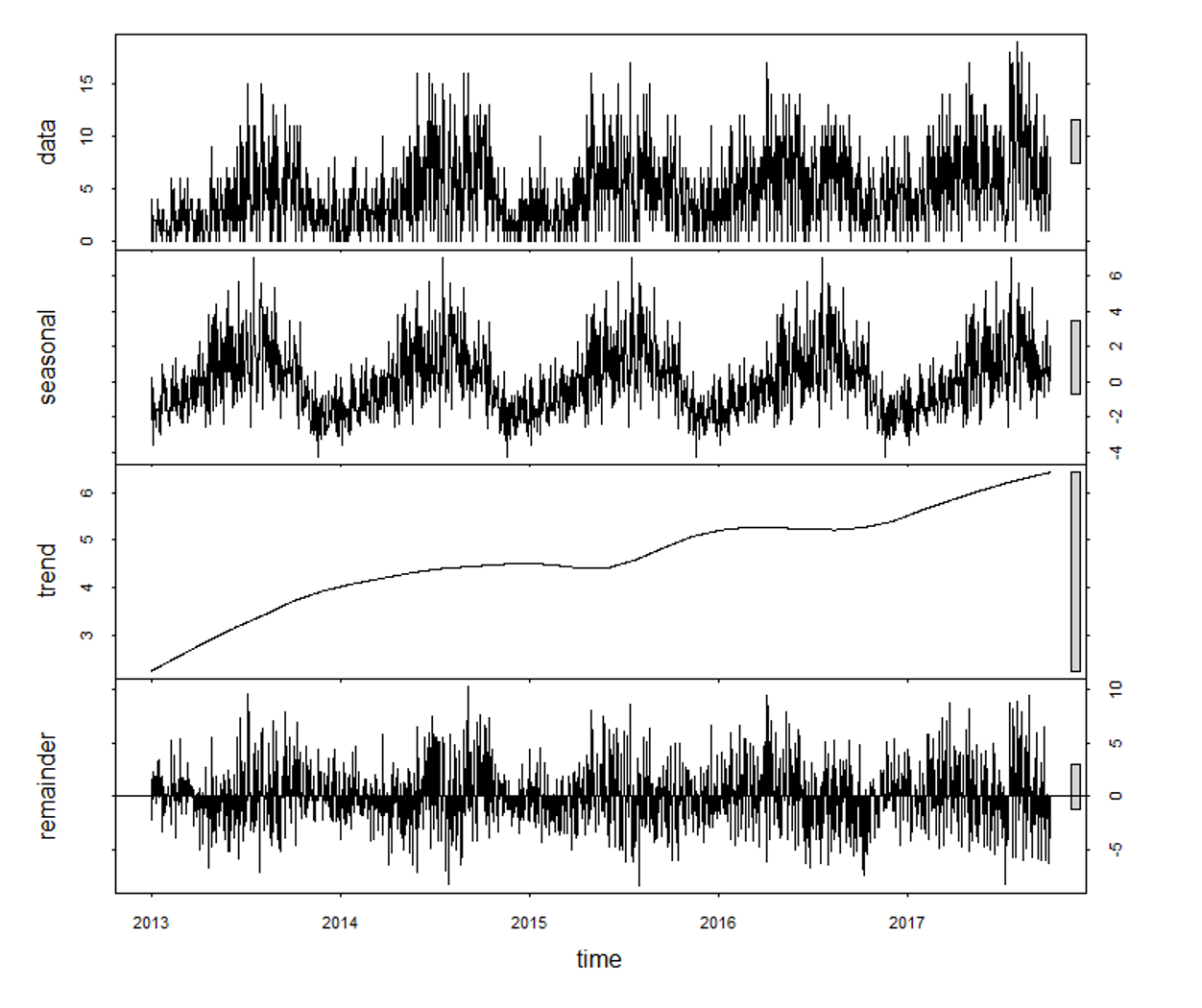


**Figure S2:** Time-series plots of clinical visits for childhood HDM-ARD for seasonality and trend from 2013 to 2017.


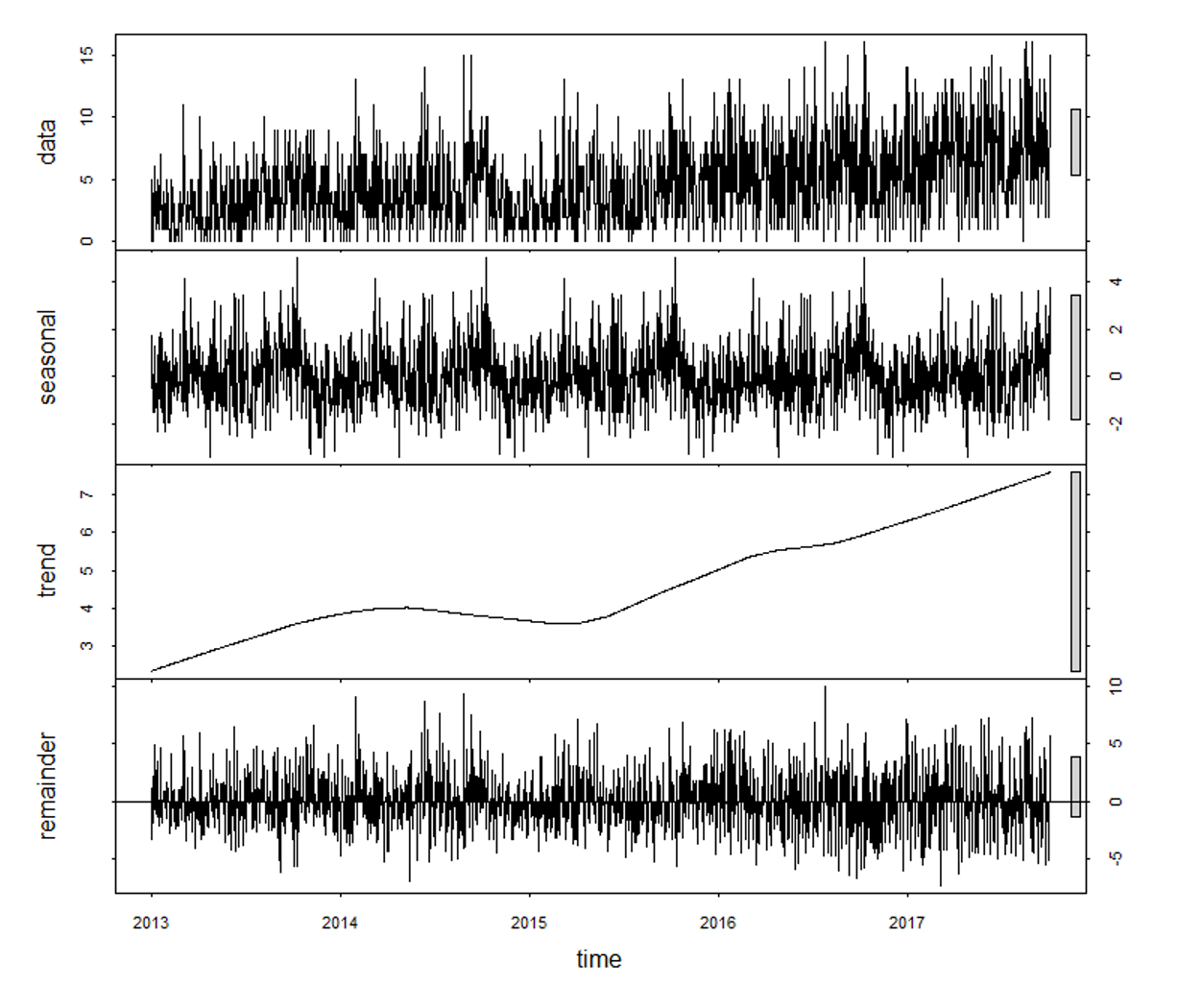
 **Figure S3:** Time-series plots of clinical visits for childhood NHDM-ARD for seasonality and trend from 2013 to 2017.


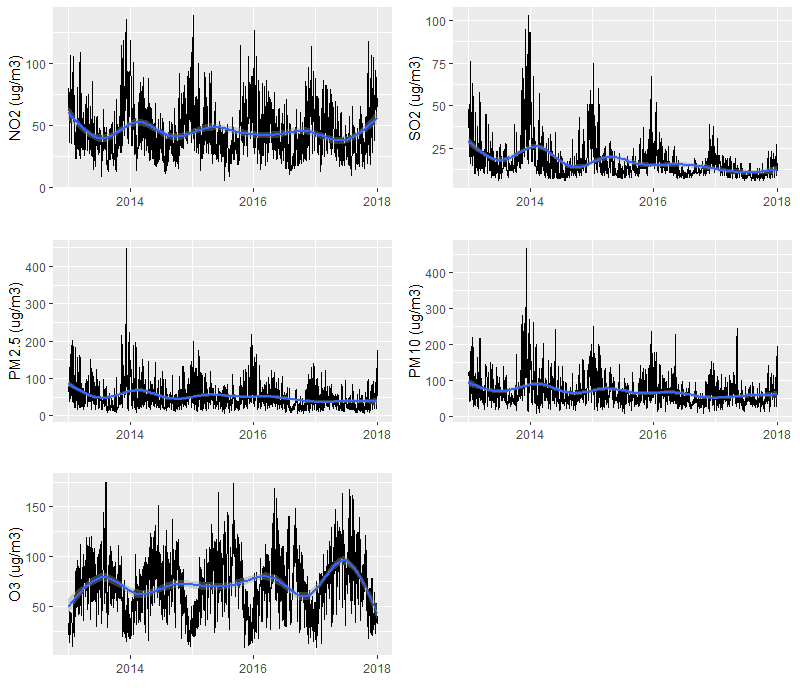
 **Figure S4:** The distribution of air pollutants from 2013 to 2017. NO_2_: nitrogen dioxide; PM_2.5_: particulate matter less than 2.5μm in aerodynamic diameter; O_3_: ozone; SO_2_: sulfur dioxide; PM_10_: particulate matter less than 10μm in aerodynamic diameter. Blue smoothed lines were superimposed on each graph to present the long-term trends.


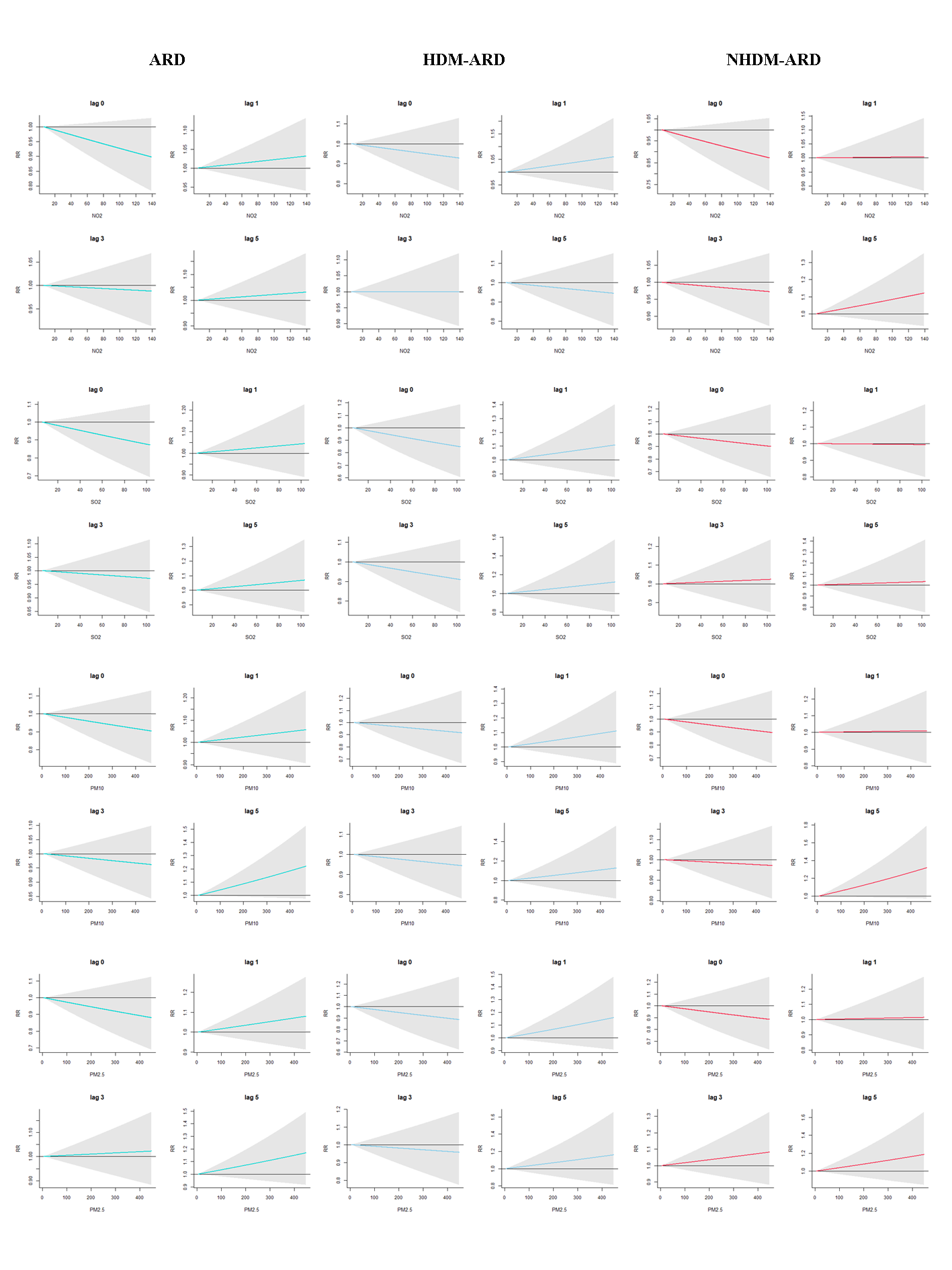
 **Figure S5** The overall exposure-response association between NO2, SO2, PM10, PM2.5 and daily clinical visits for the single-day effects of childhood ARD, HDM-ARD and NHDM-ARD. RR: relative risk; ARD: allergic respiratory disease; HDM-ARD: allergic respiratory disease induced by house dust mite; NHDM-ARD: allergic respiratory disease induced by non-house dust mite; Green color indicates childhood ARD; Blue color represents childhood ARD; Red color means childhood NHDM-ARD.
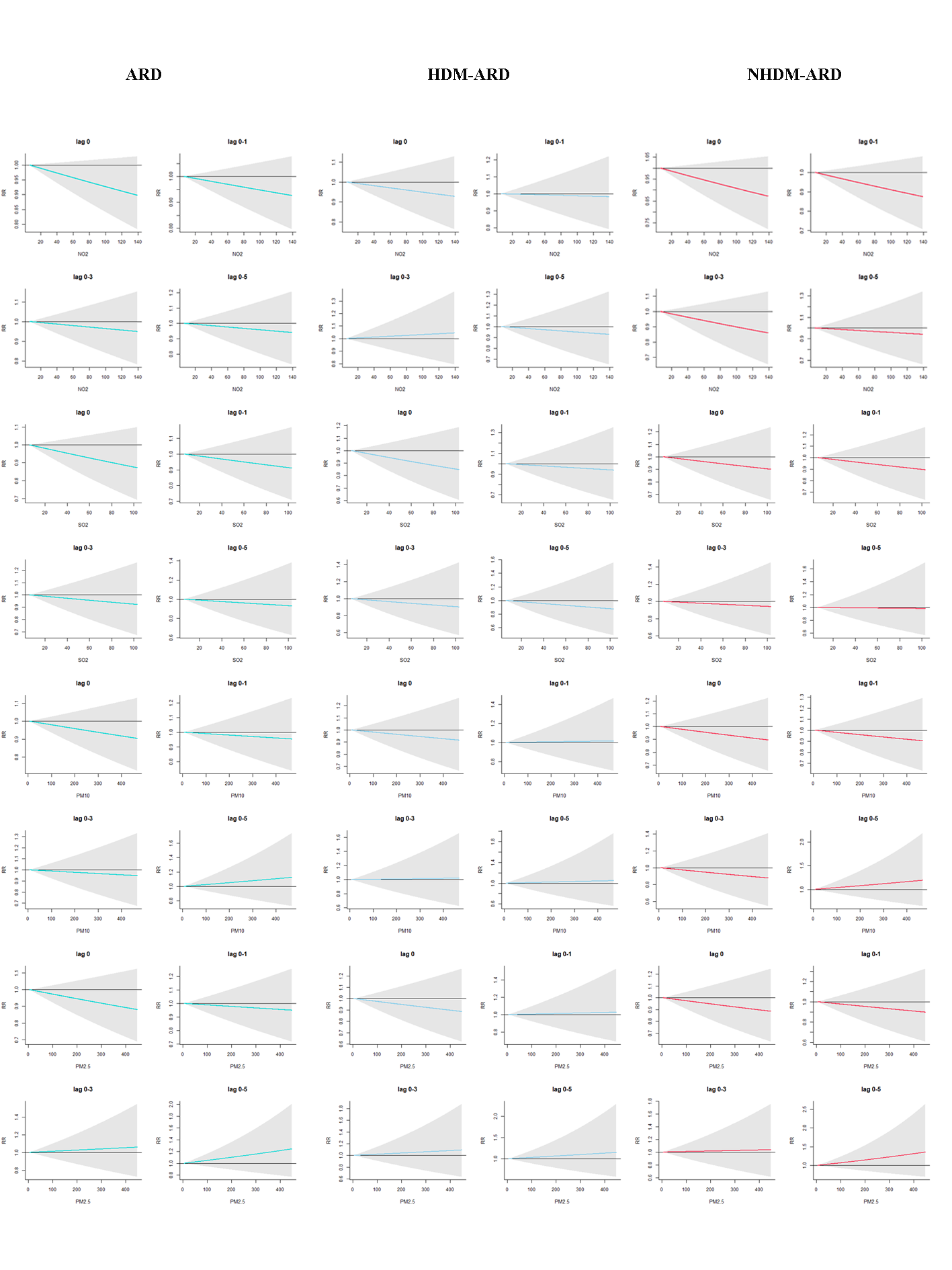
 **Figure S6** The overall exposure-response association between NO2, SO2, PM10, PM2.5 and daily clinical visits for the cumulative lagged effects of childhood ARD, HDM-ARD and NHDM-ARD. RR: relative risk; ARD: allergic respiratory disease; HDM-ARD: allergic respiratory disease induced by house dust mite; NHDM-ARD: allergic respiratory disease induced by non-house dust mite; Green color indicates childhood ARD; Blue color represents childhood ARD; Red color means childhood NHDM-ARD.


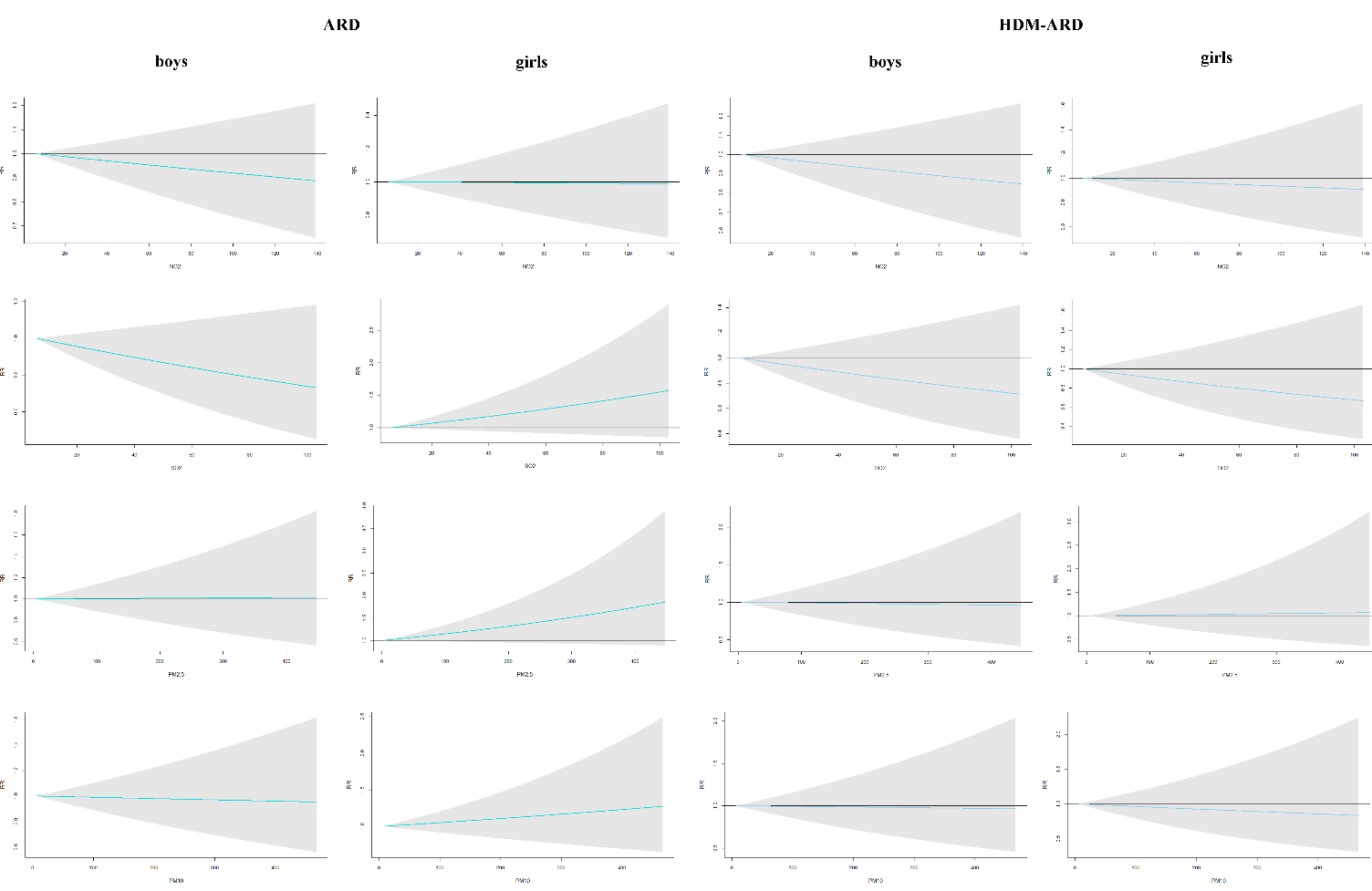


**Figure S7** The overall exposure-response association between NO2, SO2, PM10, PM2.5 and daily clinical visits for childhood ARD and HDM-ARD based on different genders. RR: relative risk; ARD: allergic respiratory disease; HDM-ARD: allergic respiratory disease induced by house dust mite; Green color indicates childhood ARD; Blue color represents childhood ARD.


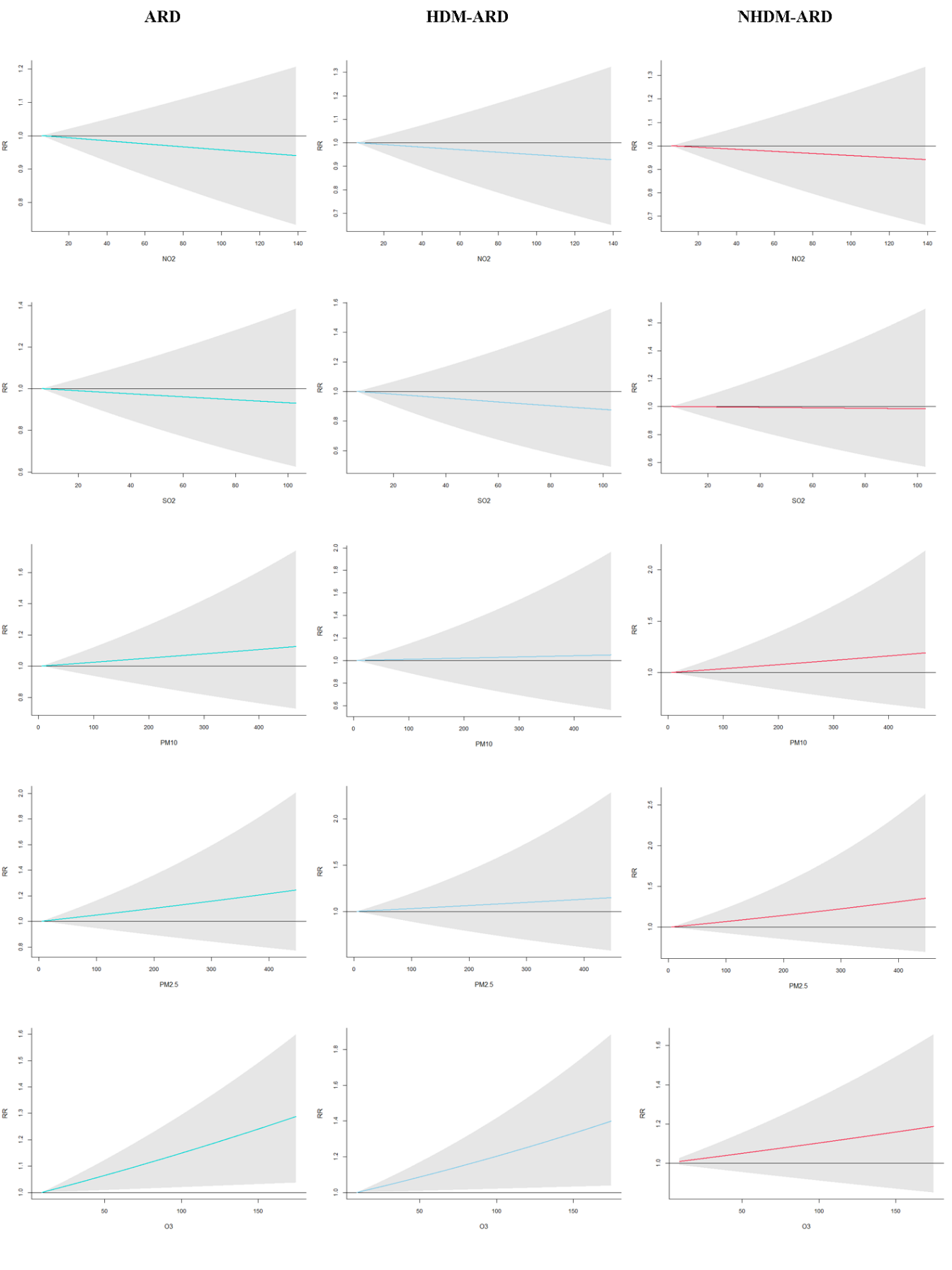
 **Figure S8** The overall exposure-response relationships between air pollutants and clinical visits for childhood ARD, HDM-ARD and NHDM-ARD. RR: relative risk; ARD: allergic respiratory disease; HDM-ARD: allergic respiratory disease induced by house dust mite; NHDM-ARD: allergic respiratory disease induced by non-house dust mite; Green color indicates childhood ARD; Blue color represents childhood ARD; Red color means childhood NHDM-ARD.


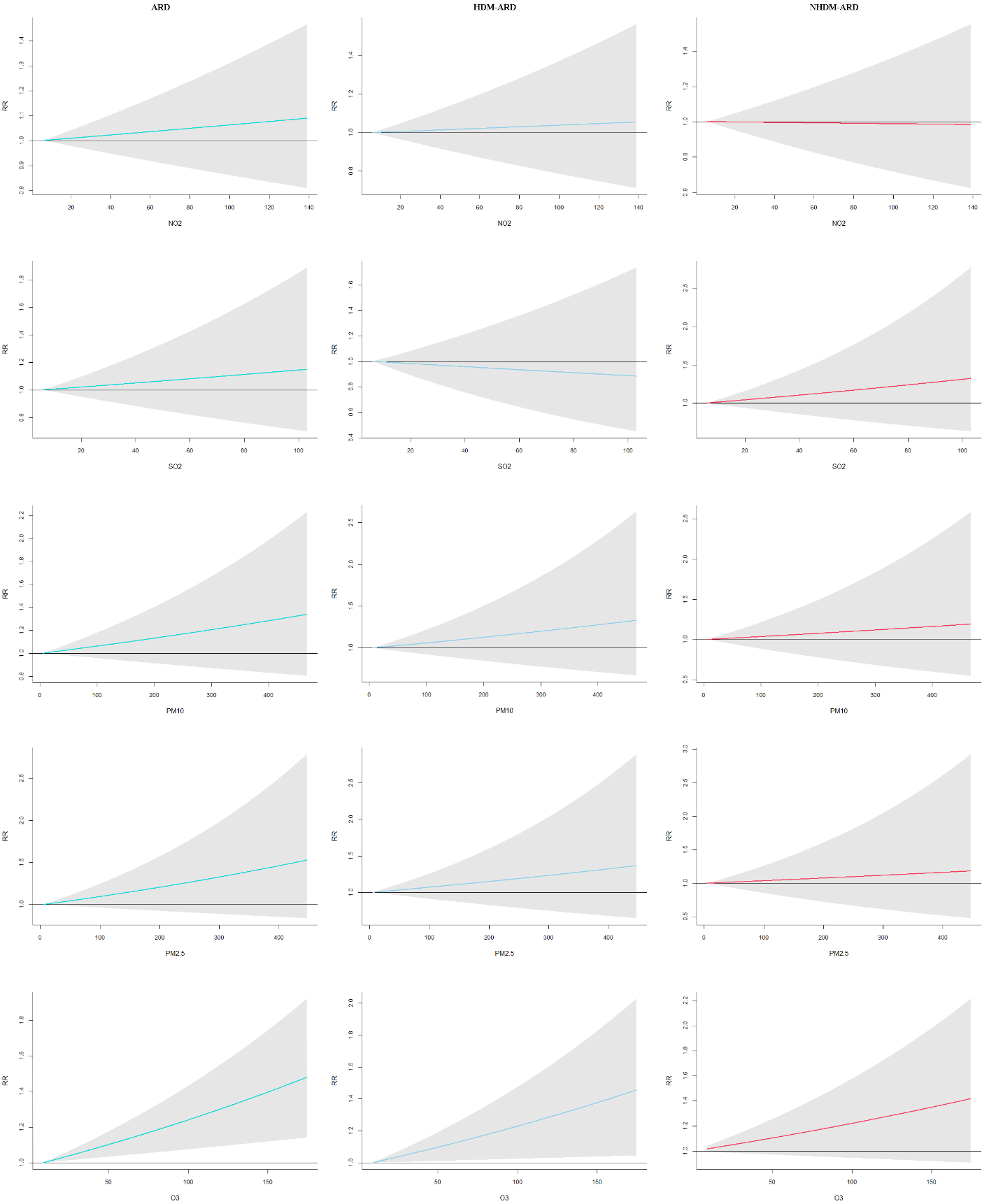
 **Figure S9** The overall exposure-response relationships between air pollutants and outpatient visits for childhood ARD, HDM-ARD and NHDM-ARD. RR: relative risk; ARD: allergic respiratory disease; HDM-ARD: allergic respiratory disease induced by house dust mite; NHDM-ARD: allergic respiratory disease induced by non-house dust mite; Green color indicates childhood ARD; Blue color represents childhood ARD; Red color means childhood NHDM-ARD.


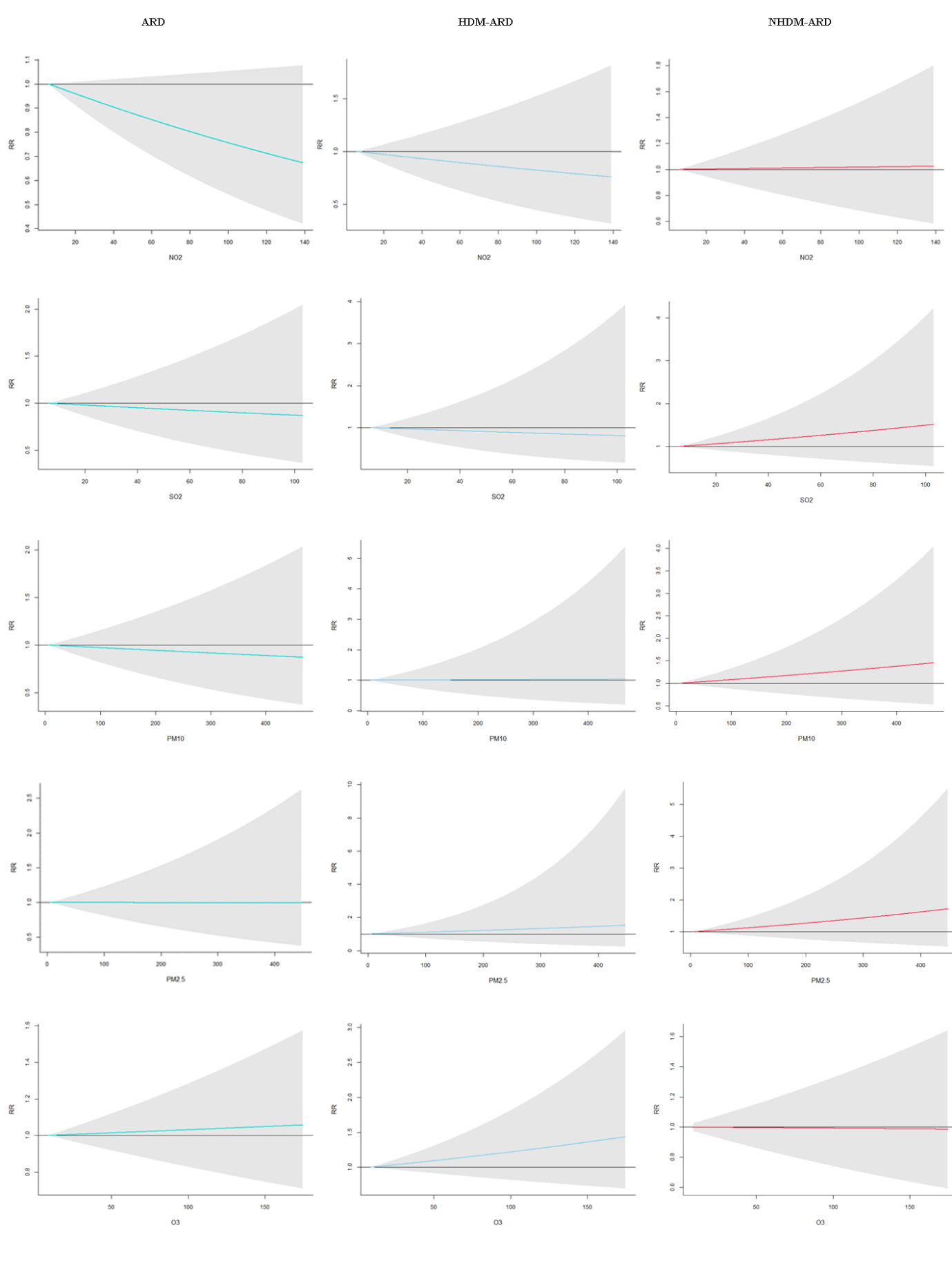
 **Figure S10** The overall exposure-response relationships between air pollutants and inpatient visits for childhood ARD, HDM-ARD and NHDM-ARD. RR: relative risk; ARD: allergic respiratory disease; HDM-ARD: allergic respiratory disease induced by house dust mite; NHDM-ARD: allergic respiratory disease induced by non-house dust mite; Green color indicates childhood ARD; Blue color represents childhood ARD; Red color means childhood NHDM-ARD.

**Table S.1** Distribution of daily clinical visits for childhood ARD, HDM-ARD, NHDM-ARD and air pollutants from 2013 to 2017

| variables | mean | min | P_25_ | P_50_ | P_75_ | max | IQR |
| --- | --- | --- | --- | --- | --- | --- | --- |
| Daily clinical visits |  |  |  |  |  |  |  |
| ARD | 9 | 0 | 5 | 9 | 13 | 31 | 8 |
| HDM-ARD | 5 | 0 | 2 | 4 | 7 | 19 | 5 |
| NHDM-ARD | 5 | 0 | 2 | 4 | 7 | 16 | 5 |
| Air pollutants |  |  |  |  |  |  |  |
| NO_2_ (μg/m^3^) | 45.4 | 6.0 | 30.0 | 41.0 | 56.0 | 139.0 | 26.0 |
| SO_2_ (μg/m^3^) | 16.9 | 6.0 | 10.0 | 13.0 | 19.0 | 103.0 | 9.0 |
| PM_10_ (μg/m^3^) | 67.3 | 6.0 | 40.0 | 56.0 | 82.0 | 467.0 | 42.0 |
| PM_2.5_ (μg/m^3^) | 49.9 | 5.0 | 26.0 | 41.0 | 63.0 | 447.0 | 37.0 |
| O_3_ (μg/m^3^) | 72.1 | 9.0 | 51.0 | 72.0 | 91.6 | 174.8 | 40.6 |

Values of PM_10_, PM_2.5_, NO_2_ and SO_2_ were daily hourly average; Values of O_3_ were computed using 1-h mean concentrations; ARD: allergic respiratory disease; HDM-ARD: allergic respiratory disease induced by house dust mite; NHDM-ARD: allergic respiratory disease induced by non-house dust mite; NO_2_: nitrogen dioxide; SO_2_: sulfur dioxide; PM_10_: particulate matter less than 10μm in aerodynamic diameter; PM_2.5_: particulate matter less than 2.5μm in aerodynamic diameter; O_3_: ozone; min: minimum; max: maximum; IQR: interquartile range; P_25_, P_50_ and P_75_ indicate the 25^th^, 50^th^, and 75^th^ percentile of the value, respectively.

**Table S.2** Spearman correlation coefficients between environmental factors during 2013–2017.

| Variables | ARD | HDM-ARD | N-HDM-ARD | Tmean | NO_2_ | PM_10_ | PM_2.5_ | SO_2_ |
| --- | --- | --- | --- | --- | --- | --- | --- | --- |
| HDM-ARD | **0.88** |  |  |  |  |  |  |  |
| NHDM-ARD | **0.85** | **0.52** |  |  |  |  |  |  |
| Mean temp | **0.21** | **0.29** | 0.05 |  |  |  |  |  |
| NO_2_ | -0.05 | **-0.09** | 0.02 | **-0.48** |  |  |  |  |
| PM_10_ | **-0.14** | **-0.15** | **-0.09** | **-0.25** | **0.66** |  |  |  |
| PM_2.5_ | **-0.18** | **-0.19** | **-0.12** | **-0.28** | **0.71** | **0.89** |  |  |
| SO_2_ | **-0.22** | **-0.22** | **-0.16** | **-0.48** | **0.69** | **0.76** | **0.72** |  |
| O_3_ | 0.05 | **0.08** | 0.00 | **0.47** | **-0.43** | -0.04 | **-0.10** | **-0.26** |

ARD: allergic respiratory disease; HDM-ARD: allergic respiratory disease induced by house dust mite; NHDM-ARD: allergic respiratory disease induced by non-house dust mite; Tmean: daily mean temperature; NO_2_: nitrogen dioxide; SO_2_: sulfur dioxide; PM_10_: particulate matter less than 10 μm in aerodynamic diameter; PM_2.5_: particulate matter less than 2.5μm in aerodynamic diameter; O_3_: ozone. Bold values are statistically significant (p < 0.05).
